# Targeted therapy against Bcl-2-related proteins in breast cancer cells

**DOI:** 10.1186/bcr1323

**Published:** 2005-09-28

**Authors:** Manabu Emi, Ryungsa Kim, Kazuaki Tanabe, Yoko Uchida, Tetsuya Toge

**Affiliations:** 1 Department of Surgical Oncology, Research Institute for Radiation Biology and Medicine, Hiroshima University, Hiroshima, Japan; 2 International Radiation Information Center, Research Institute for Radiation Biology and Medicine, Hiroshima University, Hiroshima, Japan

## Abstract

**Introduction:**

Bcl-2 and Bcl-xL confer resistance to apoptosis, thereby reducing the effectiveness of chemotherapy. We examined the relationship between the expression of Bcl-2 and Bcl-xL and chemosensitivity of breast cancer cells, with the aim of developing specific targeted therapy.

**Methods:**

Four human breast cancer cell lines were examined, and the effects of antisense (AS) *Bcl-2 *and AS *Bcl-xL *phosphorothioate oligodeoxynucleotides (ODNs) on chemosensitivity were tested *in vitro *and *in vivo*. Chemosensitivity was evaluated by the MTT (3-(4,5-dimethylthiazol-2-yl)-2,5-diphenyl-2*H*-tetrazolium bromide) assay, and the antitumor effect was assessed *in vivo *by the success of xenograft transplantation into athymic mice.

**Results:**

Treatment with AS *Bcl-2 *and *Bcl-xL *ODNs resulted in a sequence-specific decrease in protein expression, compared with controls. Treatment of BT-474, ZR-75-1, and MDA-MB-231 cells with AS *Bcl-2 *increased chemosensitivity to doxorubicin (DOX), mitomycin C (MMC), paclitaxel (TXL), and docetaxel (TXT). Transfection of the *Bcl-2 *gene into MDA-MB-453 cells decreased sensitivity to DOX and MMC. Treatment of MDA-MB-231, BT-474, and ZR-75-1 cells with AS *Bcl-xL *increased chemosensitivity to DOX, MMC and taxanes to a smaller extent than AS *Bcl-2*. This occurred in the setting of increased Bax and cleaved poly(ADP-ribose) polymerase, as well as decreased Bcl-2 and pAkt. AS *Bcl-2 *ODNs induced splenomegaly in association with increased serum IL-12, which was attenuated by methylation of the CpG motifs of AS *Bcl-2*; however, methylated CpG failed to negate the increased antitumor effect of AS *Bcl-2*. Bcl-2 and Bcl-xL, to a smaller extent, are major determinants of chemosensitivity in breast cancer cells.

**Conclusion:**

Targeted therapy against Bcl-2 protein with the use of AS ODNs might enhance the effects of chemotherapy in patients with breast cancer.

## Introduction

Bcl-2 and Bcl-xL proteins are inhibitors of the mitochondrial apoptosis pathway; they exert their action by blocking their proapoptotic counterparts, including Bid and Bax, thereby preventing the release of cytochrome *c *and the activation of caspase [1,2]. Bcl-xL shows remarkable homology to Bcl-2 and inhibits apoptosis as effectively as Bcl-2 in some cells. Furthermore, Bcl-xL is capable of preventing cell death when Bcl-2 fails to do so, suggesting that these proteins exert independent effects on the mitochondrial apoptotic pathway [3]. Given that Bcl-2 and Bcl-xL are capable of inhibiting anticancer drug-induced apoptosis, which is mediated by the voltage-dependent anion channel (VDAC) in the outer mitochondrial membrane, overexpression of Bcl-2 and Bcl-xL might confer resistance to chemotherapy [4]. In fact, overexpression of Bcl-2 and Bcl-xL is observed in several cancers, including hematologic malignancies, as well as a range of solid tumors, including nasopharyngeal, colorectal, prostate, and breast cancer [5-7].

Antisense oligodeoxynucleotides (AS ODNs) are short, synthetic stretches of DNA that hybridize with specific mRNA strands corresponding to target genes. By binding to mRNA, AS ODNs prevent the translation of target proteins, thereby blocking gene expression. Phosphorothioate ODNs, in which the oxygen atom of the phosphodiester moiety of the DNA backbone is replaced by sulphur, are the most commonly used first-generation AS ODNs because they have acceptable physical and chemical properties while showing resistance to nucleases [8]. Several studies indicate that overexpression of Bcl-2 inhibits apoptosis induced by anticancer drugs, radiation, and other DNA-damaging agents [9,10]. In addition, increased sensitivity to anticancer drugs after treatment with AS Bcl-2 is observed in solid tumors, such as breast, prostate, lung, and gastric cancer [11-14]. Similarly, downregulation of Bcl-xL protein expression by AS ODNs in various tumor cell lines resulted in activation of apoptosis, as well as decreased cellular proliferation and increased sensitivity to cytotoxic chemotherapeutic agents [15,16].

The two CpG motifs of AS *Bcl-2*, which are unmethylated dinucleotide sequences of cytosine followed by guanine, are associated with potent immune stimulation [17]. CpG ODNs administered in the vicinity of various animal tumors show marked antitumor activity [18,19]. However, it remains unclear whether immune stimulation is responsible for the antitumor effects of AS *Bcl-2 *ODNs.

In the present study we examined the effects of downregulation of Bcl-2 and Bcl-xL on the chemosensitivity of breast cancer cells *in vitro *and *in vivo *with the aim of using this approach as a specific targeting therapy. The possibility of using growth inhibition as a mechanism by which AS *Bcl-2 *ODNs enhance chemosensitivity was also explored. Furthermore, we studied the effect of *Bcl-2 *gene transfection on the chemosensitivity of a breast cancer cell line that normally expresses a low basal level of Bcl-2. Finally, we attempted to evaluate the effects of the two AS *Bcl-2 *CpG motifs on immunostimulatory function and antitumor activity in athymic mice.

## Materials and methods

### Materials

The human breast cancer cell lines BT-474, ZR-75-1, MDA-MB-231, and MDA-MB-453 were obtained from the American Type Culture Collection (Manassas, VA, USA). Cells were cultured in RPMI-1640 (Gibco BRL, New York, USA) containing 10% heat-inactivated fetal bovine serum and antibiotics. Cultures were maintained in a humidified incubator at 5% CO_2 _and 37°C.

### ODNs and anticancer drugs

Phosphorothioate ODNs, purified by reverse-phase high-performance liquid chromatography, were purchased from Biologica (Tokyo, Japan). The following AS *Bcl-2 *oligonucleotide sequence, corresponding to the first six codons of the human Bcl-2 open reading frame, was used: AS 5'-TCTCCCAGCGTGCGCCAT-3' [20]. The following Bcl-2 oligonucleotide sequences were used as controls: 5'-TCTCCCAGCATGTGCCAT-3' as a two-base mismatch control (MM), and 5'-TACCGCGTGCGACCCTCT-3' as a random control (RC). Phosphorothioate oligonucleotides corresponding to the initiation site of human *Bcl-2 *described above were made with 5'-methylation of cytosine (m5C) residues in the two CpG motifs: 5'-TCTCCCAG^m5^CGTG^m5^CGCCAT-3'. The following *Bcl-xL *target sequence was used: 5'-CCATCCCGGAAGAGTTCATT-3'. In addition, the following *Bcl-xL *sequences were used as a sense control and a two-base MM: 5'-AATGAACTCTTCCGGGATGG-3' [16] and 5'-CCATCCCAGAAGAGTTTATT-3', respectively. The *Bcl-2 *and *Bcl-xL *sequences were not homologous with *Bcl-xS *or with any other known human gene sequences. All of the oligonucleotides were diluted to a concentration of 1 mM, filter-sterilized, and stored at -30°C in distilled water. Doxorubicin (DOX) and mitomycin C (MMC) were from Kyowa Hakko Co., Ltd (Tokyo, Japan), paclitaxel (TXL) was from Bristol-Myers K.K. (Tokyo, Japan), and docetaxel (TXT) was from Aventis Pharma (Tokyo, Japan). DOX, MMC, and TXT were prepared with saline solution, and TXL was dissolved in dimethyl sulfoxide (DMSO).

### Cell extraction and Western blotting

Cells were washed twice with PBS, centrifuged at 2,700 g and 4°C, and lysed with lysis buffer containing 10 mM Tris-HCl pH 8.0, 0.15 M NaCl, 1 mM EDTA, 10 mM CHAPS, 10 μg/ml aprotinin, and 0.02 mM phenylmethylsulfonyl fluoride. Lysate was incubated for 15 min on ice and centrifuged for 15 min at 2,700 g. Supernatant was collected and the protein quantity was estimated with Bio-Rad protein assay dye (Bio-Rad, Hercules, CA, USA). Samples containing equal amounts of protein (15 μg) were subjected to electrophoresis on a 12.5% sodium dodecyl sulfate-polyacrylamide gel, and transferred to a poly(vinylidene difluoride) membrane. After being blocked overnight with PBS containing 5% nonfat milk powder, the membrane was incubated with primary antibody (1:200 dilution) for 1 hour at room temperature at 25°C. Antibodies used for specific immune blotting included anti-Bcl-2, anti-Bcl-xL, anti-Bax, anti-pAkt, anti-poly(ADP-ribose) polymerase (anti-PARP), and anti-β-actin. All antibodies were obtained from Santa Cruz Biotechnology (Santa Cruz, CA, USA). The membrane was washed three times with PBS and then incubated with anti-rabbit or anti-mouse IgG antibody (1:1,000 dilution; Sigma Chemical, St Louis, MO, USA) for 1 hour at room temperature. After three washes with PBS, specific protein bands were detected with an enhanced chemiluminescence western blot detection system (ECL; Amersham Pharmacia Biotech, Little Chalfont, Bucks, UK), and detected after exposure to Hyper film ECL (Amersham Pharmacia Biotech, Little Chalfont, Bucks, UK). Each protein signal was quantified with Scion image software (Scion Corporation, Frederick, MA, USA).

### MTT assays and cell viability

Cells were seeded into 96-well plates at 10^4 ^viable cells per well and left to attach to the plate for 24 hours. After 24 hours, cells were treated with anticancer drugs for 48 hours. The final volume was 200 μl per well. Subsequently, 200 μl of medium containing 0.25 mg/ml 3-(4,5-dimethylthiazol-2-yl)-2,5-diphenyl-2*H*-tetrazolium bromide (MTT; Sigma Chemical, Tokyo, Japan) was added to each well for 3 hours. The medium was then removed and 150 μl of DMSO (Wako Pure Chemical Industries, Osaka, Japan) was added to each well for 30 min at room temperature. The absorbance of each well was measured with a microculture plate reader at 540 nm. Growth inhibition was expressed as a ratio of the mean absorbance of drug-treated cells to that of control cells. Experiments were performed in triplicate, and growth inhibition rates and IC_50 _values were calculated. Cell viability was also assessed with the trypan blue dye-exclusion test.

### Transfection

pZip_neo _plasmid expression vectors containing human *bcl-2 *cDNA were used [21]. MDA-MB-453 cells in the exponential phase of growth were transfected with the pZip_neo _plasmid expression vector with the use of Lipofectamine 2000 (Invitrogen Corp., Carlsbad, CA, USA), in accordance with the instructions provided by the manufacturer. After this, cells were selected with 400 μg/ml Geneticin Liquid (Gibco BRL, New York, USA), and the resulting clones were pooled. The expression of Bcl-2 by MDA-MB-453 cells was determined by Western blotting with a monoclonal antibody against Bcl-2.

### Antitumor activity *in vivo*

Four-week-old female athymic mice (Balb/c, nu/nu) were obtained from Clea Japan (Tokyo, Japan). The research protocol was in accordance with the institutional guidelines of the Hiroshima University Animal Care and Use Committee. BT-474 (estrogen receptor (ER)-positive), ZR-75-1 (ER-positive), and MDA-MB-231 (ER-negative), cells were established as subcutaneous xenografts by the injection of 10^7 ^cells suspended in 150 μl of Matrigel (Becton Dickinson Labware, Bedford, MA, USA) in the lateral back region.

Mice were implanted subcutaneously with pellets engineered to give a controlled release of 0.72 mg of 17β-estradiol over 60 days (Innovative Research, Sarasota, FL, USA) 1 week before estrogen-dependent cell implantation. When palpable tumors arose, three or four pieces (1 to 2 mm^3^) of nonnecrotic tissue were subcutaneously transplanted into other mice with the use of a biomedical stainless steel needle (implant needle) under anesthesia. Once tumors had reached a volume of about 100 mm^3^, mice were randomized to receive one of the following treatments: no treatment (control group); treatment with AS *Bcl-2 *or AS *Bcl-xL *ODNs; treatment with various anticancer agents; or combined treatment with AS ODNs and various anticancer agents. AS *Bcl-2 *and AS *Bcl-xL *ODNs (5 mg/kg) were administered by intraperitoneal injection 6 days a week, every other week for 4 weeks. The anticancer agents were administered once a week for 4 weeks as 10 mg/kg TXL or TXT, or 2 mg/kg MMC, by intraperitoneal injection, or 6 mg/kg doxorubicin by bolus injection into the tail vein. Serial measurements of tumor diameter were made with calipers, and tumor volumes were calculated as volume = width^2 ^× length/2.

### Serum levels of IL-12

Changes in serum levels of IL-12 after exposure to AS *Bcl-2 *ODNs, as well as AS *Bcl-2 *ODNs with methylated CpG motifs (synthetic CpG AS *Bcl-2 *ODNs), were measured by enzyme-linked immunosorbent assay (SRL, Tokyo, Japan); the results were compared with control values. Changes in splenic weight and serum IL-12 levels were evaluated after treatment with AS *Bcl-2 *or synthetic CpG AS *Bcl-2 *ODNs.

### Statistical analysis

All of the linear regression was performed with Microsoft Excel (Seattle, WA, USA). Student's *t*-test was used to measure statistical significance between two treatment groups. Multiple comparisons were performed with a one-way analysis of variance (ANOVA). Data were considered significant if *P *< 0.05.

## Results

### Expression of Bcl-2 and Bcl-xL in breast cancer cell lines

**Figure 1:**
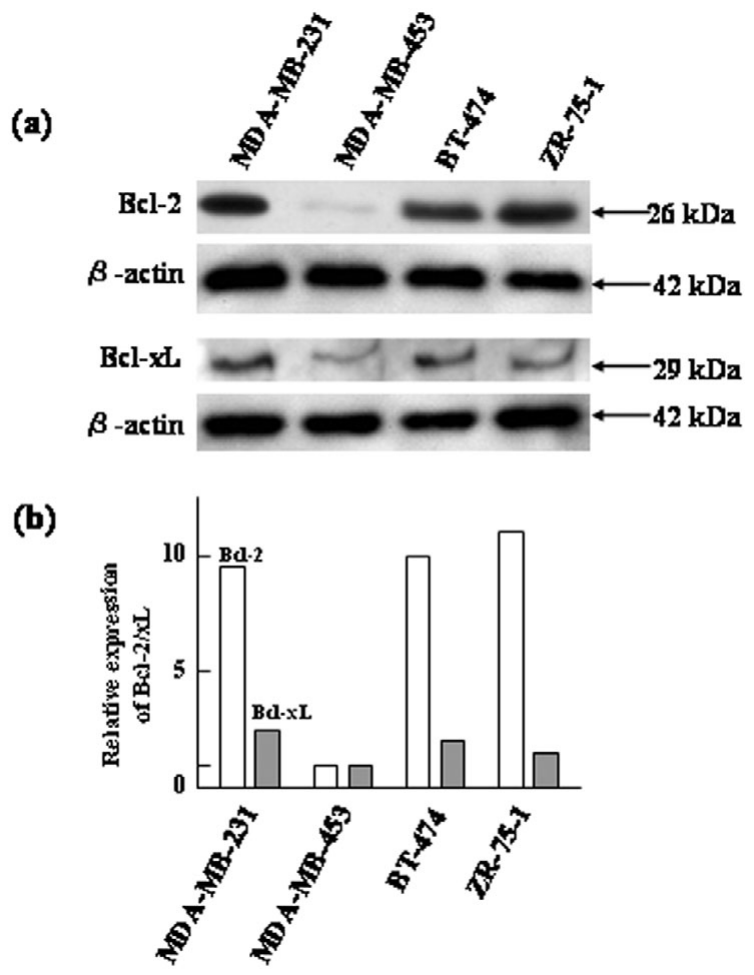
Expression levels of Bcl-2 and Bcl-xL proteins in MDA-MB-231, MDA-MD-453, BT-474, and ZR-75-1 cells. **(a) **Western blot analysis of Bcl-2 and Bcl-xL expression. **(b) **Quantification of Bcl-2 and Bcl-xL expression by densitometric analysis. The relative expression of Bcl-2 and Bcl-xL in MDA-MB-453 cells was compared with the expression in MDA-MB-231, BT-474, and ZR-75-1 cells. Results are from two representative, independent experiments.

To assess the expression of Bcl-2 and Bcl-xL in breast cancer cell lines, western blot analysis was performed (Fig. 1). Bcl-2 and Bcl-xL proteins were expressed in MDA-MB-231, BT-474, and ZR-75-1 cells, whereas Bcl-2 and Bcl-xL expression in MDA-MB-453 cells was observed to a smaller extent. These findings suggest differential regulation in the expression of Bcl-2 and Bcl-xL protein in breast cancer cells.

### Sequence-specific downregulation and cytotoxic effects of AS *Bcl-2*

**Figure 2:**
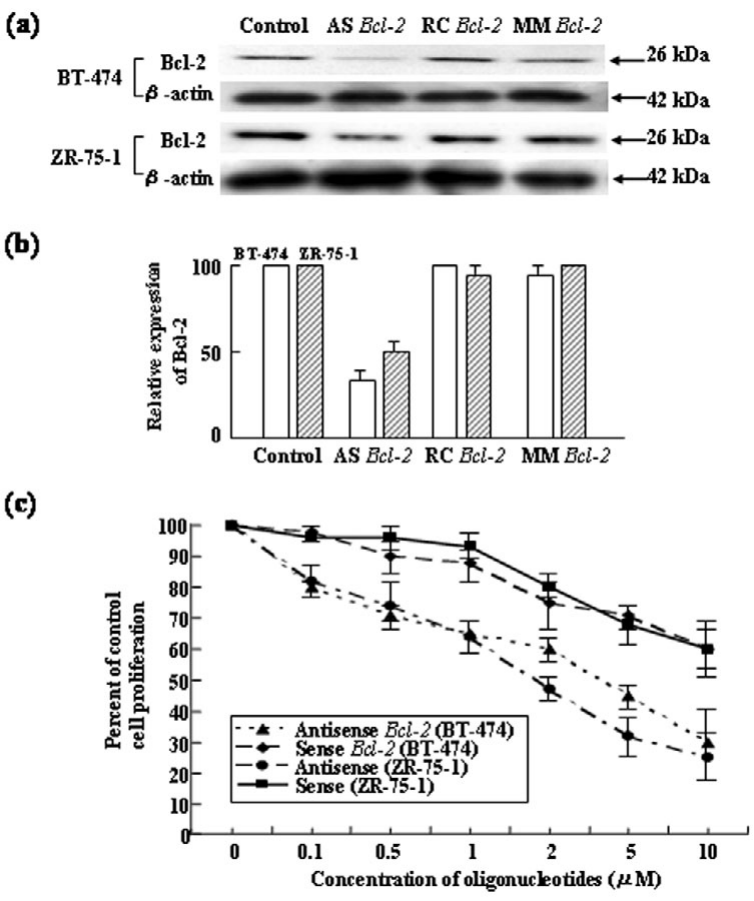
Sequence-specific downregulation and cytotoxic effects of antisense *Bcl-2 *oligodeoxynucleotides on BT-474 and ZR-75-1 cells. **(a) **Specific inhibition of Bcl-2 protein expression by treatment with antisense (AS) *Bcl-2 *oligodeoxynucleotides (ODNs). Cells were treated with 10 μg/ml Lipofectamine alone (control) or 1.0 μM AS, mismatch control (MM), or random control (RC) ODNs for 24 hours. Cells were then cultured in standard medium, total protein was extracted, and Bcl-2 and β-actin protein levels were analyzed by Western blotting. **(b) **Quantification of Bcl-2 expression by densitometric analysis. The expression of Bcl-2 in cells treated with control, AS *Bcl-2*, RC *Bcl-2*, and MM *Bcl-2 *ODNs was normalized with β-actin, and the relative values are presented. Error bars indicate SD. The data presented are from three independent experiments. **(c) **Effects of AS *Bcl-2 *ODNs on the proliferation of BT-474 and ZR-75-1 breast cancer cells *in vitro*. Cells were treated with various concentrations of AS *Bcl-2 *ODNs in 24-well dishes. Four days after treatment, cells were stained with trypan blue and counted. Error bars indicate SD. The data presented are from three independent experiments.

We examined the effect of AS oligonucleotide treatment on Bcl-2 protein expression. Western blot analysis showed that BT-474 and ZR-75-1 cells transfected with 1.0 μM AS *Bcl-2 *oligonucleotides produced significantly less Bcl-2 protein than cells treated with RC and MM oligonucleotides, or with Lipofectamine alone. Bcl-2 protein expression was decreased by 70% and 50%, compared with control values, in BT-474 and ZR-75-1 cells, respectively (Fig. 2a,b). Also, as shown in Fig. 2c, treatment of BT-474 and ZR-75-1 cells with AS *Bcl-2 *oligonucleotides inhibited cell proliferation in a dose-dependent manner. In contrast, control sense oligonucleotides had a minimal effect on cell growth at concentrations up to 1.0 μM. Greater concentrations of control sense oligonucleotides had cytotoxic effects. A dose–response effect with regard to downregulation of Bcl-2 protein expression was observed in ZR-75-1 and BT-474 cells, resulting in maximal inhibition approaching 50% and 70%, respectively, at concentrations up to 2.0 μM. However, the maximum inhibition of 70% for Bcl-2 protein was also observed at 1.0 μM AS *Bcl-2 *ODNs, which was the optimal dose for limiting cytotoxicity in the MDA-MB-231 cell line (data not shown).

### Effect of AS Bcl-2 on chemosensitivity

To evaluate whether treatment of BT-474, ZR-75-1, and MDA-MB-231 cells with AS Bcl-2 in combination with anticancer drugs enhances antitumor effects *in vitro*, we used the MTT assay to examine the efficacy of DOX, MMC, TXL, and TXT in the presence and absence of AS Bcl-2. BT-474, ZR-75-1, and MDA-MB-231 cells were treated with 1.0 μM sense Bcl-2 or AS Bcl-2, or Lipofectamine alone, for 24 hours, and then incubated with various concentrations of anticancer drugs for a further 48 hours. MTT assays were then performed to determine cell viability. As shown in Table 1, although treatment with sense Bcl-2 affected the chemosensitivity somewhat, treatment of BT-474 cells with AS Bcl-2 resulted in 8.3-fold, 8.3-fold, and 10.0-fold increases in sensitivity to DOX, TXL, and TXT, respectively. In contrast, treatment with AS Bcl-2 and MMC did not enhance the drug sensitivity of BT-474 cells to the same extent. Treatment of ZR-75-1 cells with AS Bcl-2 resulted in 7.2-fold, 2.5-fold, 3.1-fold and 3.9-fold increases in sensitivity to DOX, MMC, TXL, and TXT, respectively. Treatment of MDA-MB-231 cells with AS Bcl-2 resulted in 5.9-fold, 3.1-fold, 6.5-fold and 5.2-fold increases in sensitivity to DOX, MMC, TXL, and TXT, respectively. These results indicate that downregulation of Bcl-2 by AS Bcl-2 ODNs in breast cancer cells that normally overexpress Bcl-2 enhances their drug sensitivity, especially the sensitivity to DOX and taxanes.

### Sequence-specific downregulation and cytotoxic effects of AS *Bcl-xL*

**Figure 3:**
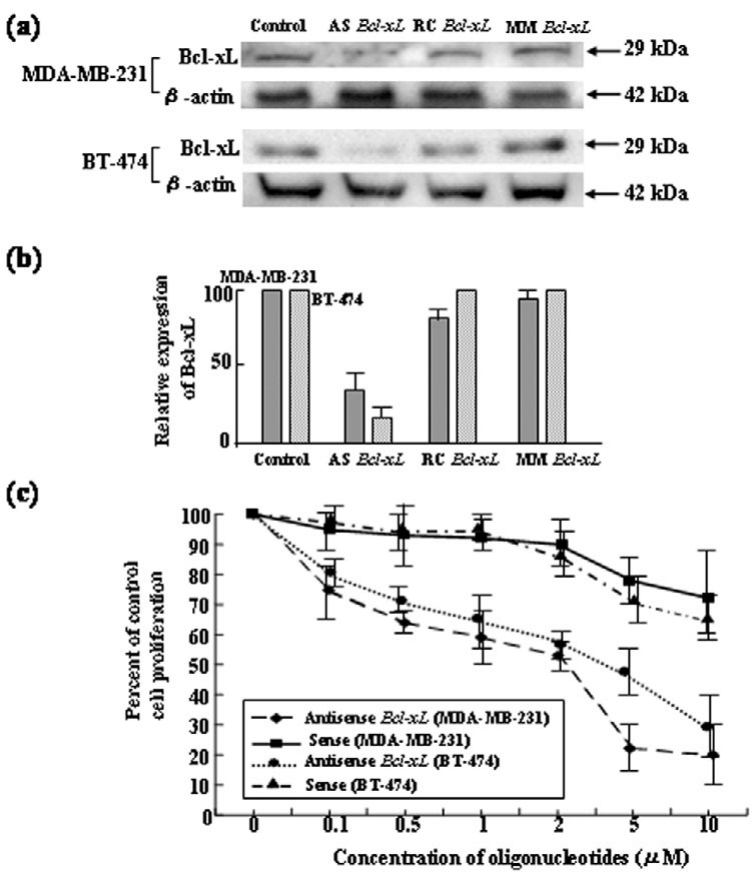
Sequence-specific downregulation and cytotoxic effects of antisense *Bcl-xL *oligodeoxynucleotides on MDA-MB-231 and BT-474 cells. **(a) **Specific inhibition of Bcl-xL protein expression by treatment with antisense (AS) *Bcl-xL *oligodeoxynucleotides (ODNs). Cells were treated with 10 μg/ml Lipofectamine alone (control) or 1.0 μM AS, mismatch control (MM), or random control (RC) ODNs for 24 hours. Cells were then cultured in standard medium, total protein was extracted, and Bcl-xL and β-actin protein levels were analyzed by Western blotting. **(b) **Quantification of Bcl-xL protein expression by densitometric analysis. The Bcl-xL protein expression was normalized with β-actin, and the relative values are presented. Error bars indicate SD. The data presented are from three independent experiments. **(c) **Effects of AS *Bcl-xL *ODNs on the proliferation of MDA-MB-231 and BT-474 breast cancer cells *in vitro*. Cells were treated with various concentrations of AS *Bcl-xL *ODNs in 24-well dishes. Four days after treatment, cells were stained with trypan blue and counted. Error bars indicate SD. The data presented are from three independent experiments.

We examined the effect of transfection with 1.0 μM AS *Bcl-xL*, mismatch, and random ODNs, on Bcl-xL protein expression in MDA-MB-231 and BT-474 cells (Fig. 3a). A 70% decrease in Bcl-xL protein expression was observed in cells treated with AS *Bcl-xL *ODNs, compared with control cells, but treatment with mismatch or random ODNs produced little change. These findings indicate that the effects of treatment with AS *Bcl-xL *are due to its specificity for *Bcl-xL *mRNA. The decrease in Bcl-xL protein expression by the treatment with AS *Bcl-xL *was confirmed by quantification by densitometric analysis (Fig. 3b). As shown in Fig. 3c, treatment with AS *Bcl-xL *ODNs resulted in dose-dependent inhibition of cell proliferation. In contrast, control sense oligonucleotides had minimal effects on cell growth at concentrations up to 2.0 μM. Greater oligonucleotide concentrations resulted in cytotoxicity. The optimal dose of AS *Bcl-xL *ODNs was determined as 1.0 μM to strike a balance between the dose–response effect and cytotoxicity. In ZR-75-1 cells, the suppression of Bcl-xL protein expression by AS *Bcl-xL *was also about 70%, and the optimal dose of AS *Bcl-xL *was 1.0 μM, in terms of limiting its cytotoxicity (data not shown).

### Effect of AS *Bcl-xL *on chemosensitivity

We examined the chemosensitivity of MDA-MB-231, BT-474, and ZR-75-1 cells to DOX, MMC, TXL, and TXT in the presence and absence of AS *Bcl-xL *with the MTT assay. As shown in Table 2, although pretreatment with sense *Bcl-xL *affected the chemosensitivity somewhat, the sensitivity to DOX, MMC, TXL, and TXT in MDA-MB-231 cells was increased 2.5-fold, 3.6-fold, 2.9-fold, and 1.9-fold, respectively, by pretreatment with AS *Bcl-xL*, as determined by increases in IC_50 _values. In addition, drug sensitivity to DOX, MMC, TXL, and TXT in BT-474 cells was increased 1.5-fold, 1.6-fold, 1.8-fold, and 2.1-fold, respectively, and drug sensitivity to DOX, MMC, TXL, and TXT in ZR-75-1 cells was increased 1.3-fold, 2.2-fold, 1.6-fold, and 1.8-fold, respectively. These results indicate that downregulation of Bcl-xL by AS *Bcl-xL *enhances drug sensitivity, but not to the same extent as downregulation of Bcl-2. This is particularly true for DOX and taxanes.

### Effect of transfection with the *bcl-2 *gene on chemosensitivity

**Figure 4:**
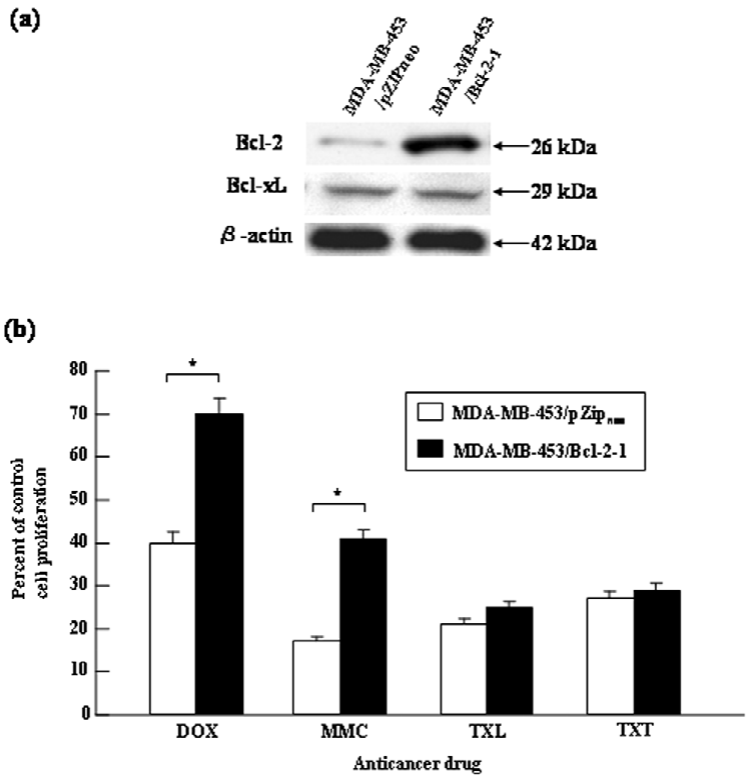
Changes in chemosensitivity of MDA-MB-453 cells to anticancer drugs after transfection with *bcl-2 *gene. **(a) **Expression levels of Bcl-2 and Bcl-xL were examined in MDA-MB-453 cells by Western blot analysis. MDA-MB-453 cells transfected with the *bcl-2 *gene expressed greater levels of Bcl-2 protein; however, similar levels of Bcl-xL protein were observed. **(b) **Cells treated with 0.15 μM doxorubicin (DOX) or with 2.0 μM mitomycin C (MMC), paclitaxel (TXL) or docetaxel (TXT). After 48 hours, cells were stained with trypan blue and counted. Each point represents the mean of triplicate experiments. Error bars indicate SD. *, *P *< 0.05, Student's *t*-test. The data presented are from three independent experiments.

To determine the effect of Bcl-2 on chemosensitivity, cell proliferation was compared between MDA-MB-453/pZip_neo _and MDA-MB-453/Bcl-2-1 cells, which were transfected with empty plasmid vector or plasmid vector encoding the *bcl-2 *gene. As shown in Fig. 4, MDA-MB-453 cells were resistant to DOX and MMC after transfection with the *bcl-2 *gene (*P *< 0.05, Student's *t*-test), whereas drug sensitivity to taxanes was not changed. Two other clones transfected with the *bcl-2 *gene had similar drug sensitivities to those of control cells transfected with vector alone (data not shown).

### Effect of AS *Bcl-2 *on expression of apoptosis-related proteins

**Figure 5:**
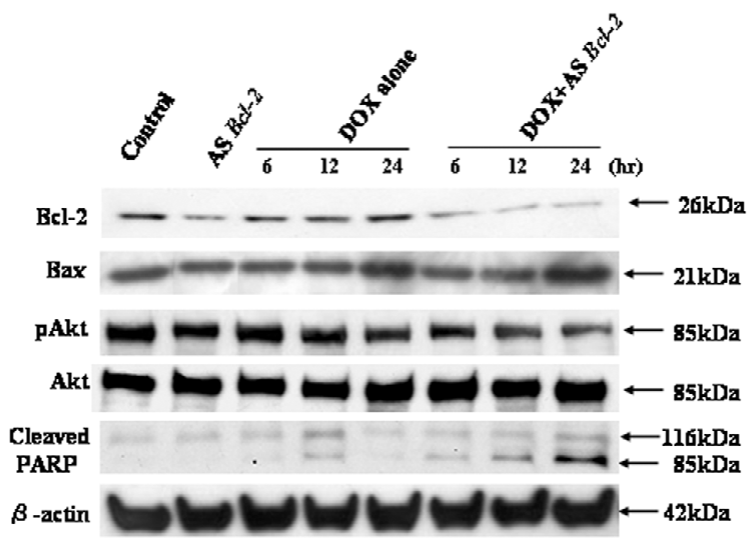
Effect of treatment of ZR-75-1 cells with antisense *Bcl-2 *and doxorubicin on the apoptosis-related proteins. Cells were pretreated with 10 μg/ml Lipofectamine alone, or 1.0 μM antisense *Bcl-2 *oligodeoxynucleotides, for 24 hours. Cells were then cultured in standard medium, after which they were treated with 0.5 μM doxorubicin (DOX) for 6, 12, or 24 hours. Whole cell lysate was then extracted and subjected to Western blotting. The data presented are from more than two independent experiments.

To investigate the effect of combined treatment with anticancer drugs and AS *Bcl-2 *on apoptosis, we analyzed the expression of apoptosis-related proteins by Western blotting. As shown in Fig. 5, treatment with AS *Bcl-2 *and DOX markedly suppressed Bcl-2 expression from that observed after treatment with DOX alone. Combined treatment with AS *Bcl-2 *and DOX enhanced the expression of Bax, which is a proapoptotic protein, and inhibited phosphorylated Akt (pAkt), which is an antiapoptotic protein. Furthermore, the cleaved PARP increased over time after combined treatment with AS *Bcl-2 *and DOX, indicating an increased rate of apoptotic cell death.

### Effect of combined treatment with AS *Bcl-2 *and various anticancer drugs *in vivo*

**Figure 6:**
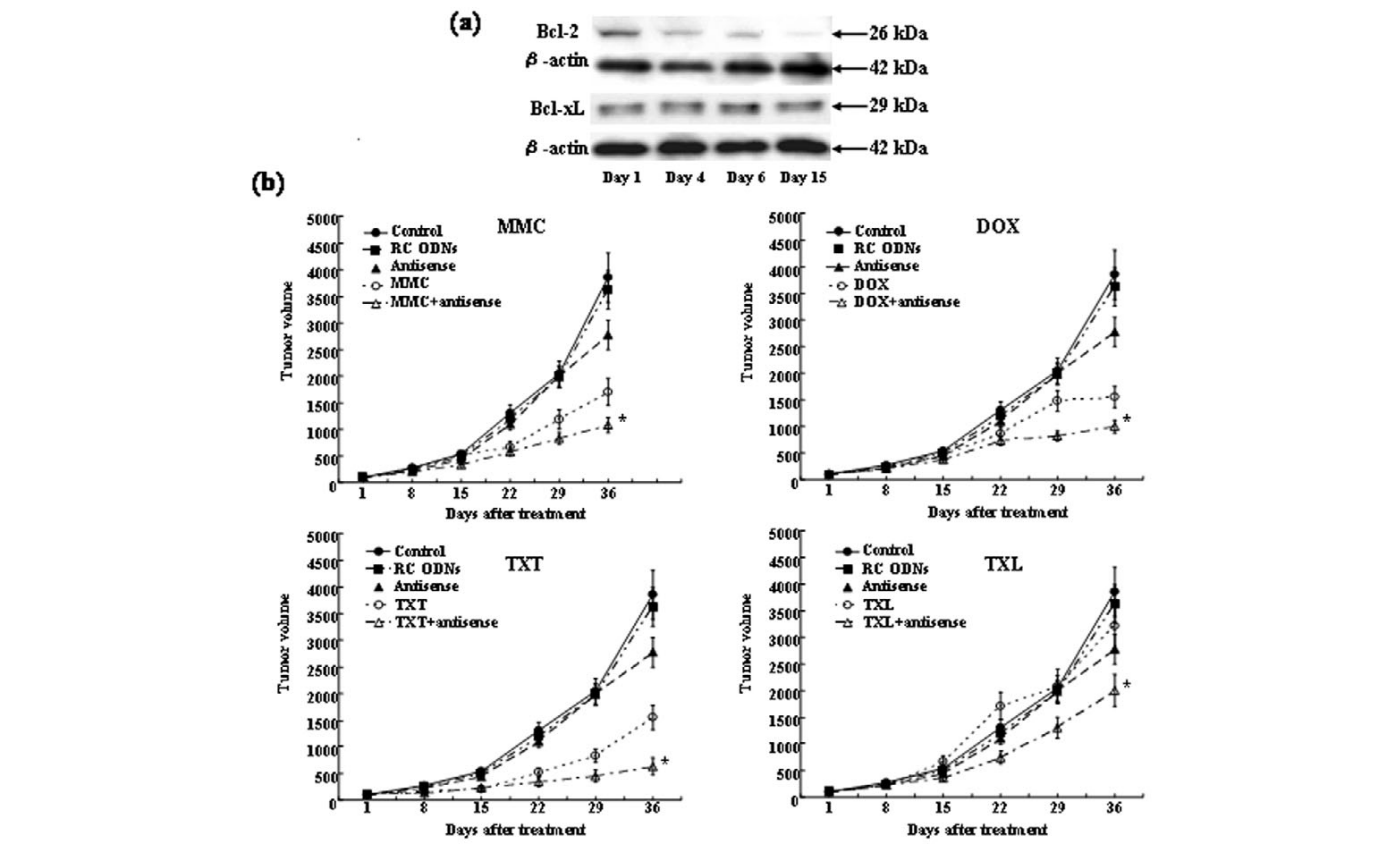
Effects of treatment with antisense *Bcl-2 *and mitomycin C, doxorubicin, paclitaxel, or docetaxel on BT-474 cells. **(a) **Expression levels of Bcl-2 and Bcl-xL protein in BT-474 cells transplanted into athymic mice after treatment with antisense (AS) *Bcl-2 *oligodeoxynucleotides (ODNs) were measured by Western blot analysis at the indicated time points. **(b) **Enhancement of the antitumor effects of anticancer drugs by AS *Bcl-2 *ODNs in BT-474 tumor xenografts. Each point represents the mean tumor volume of the eight mice in each group. Error bars indicate SD. *, *P *< 0.05, analysis of variance with Fisher's least significant difference test. The data presented are from two independent experiments. MMC, mitomycin C; DOX, doxorubicin; TXL, paclitaxel; TXT, docetaxel.

To investigate the effects of combined treatment with AS *Bcl-2 *and various anticancer agents *in vivo*, we examined tumor growth after combined treatment of BT-474 and ZR-75-1 cells transplanted into athymic mice with AS *Bcl-2 *and MMC, DOX, TXL, and TXT. On the basis of the protocols used in previous ODN-treatment studies, mice were treated with 5 mg/kg AS *Bcl-2 *and either 10 mg/kg TXL or TXT, 6 mg/kg DOX, or 2 mg/kg MMC [7,12,22]. As shown in Fig. 6a, Bcl-2 expression was suppressed on day 4, and almost completely inhibited on day 15, after treatment with AS *Bcl-2*, which was given by intraperitoneal injection every other week for 4 weeks. In contrast, Bcl-xL did not change after treatment with AS *Bcl-2*.

**Figure 7:**
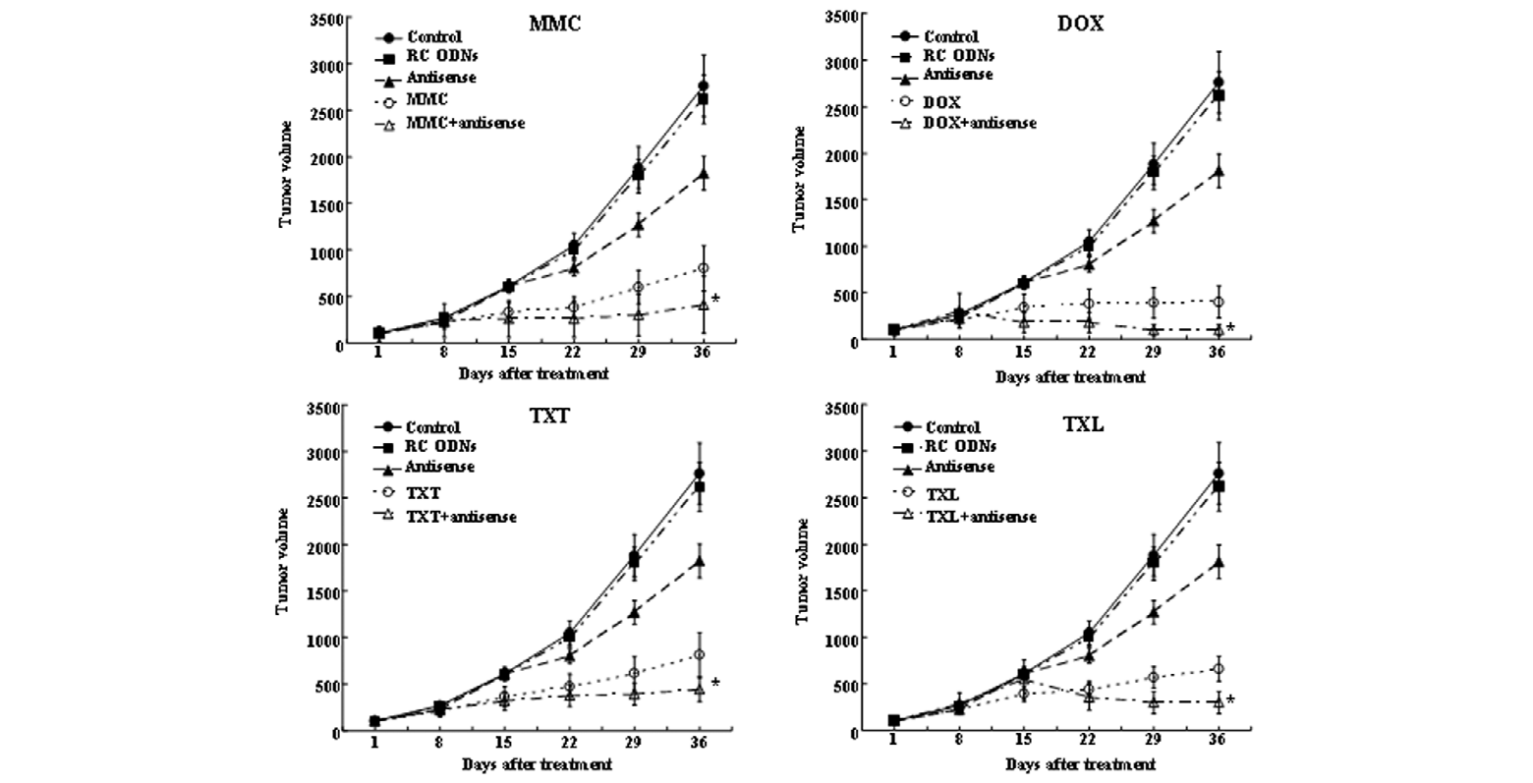
Effects of treatment with antisense *Bcl-2 *and mitomycin C, doxorubicin, paclitaxel, or docetaxel on ZR-75-1 cells. Enhancement of the antitumor effects of anticancer drugs by antisense *Bcl-2 *oligodeoxynucleotides in ZR-75-1 tumor xenografts. Each point represents the mean tumor volume of the eight mice in each group. Error bars indicate SD. *, *P *< 0.05, analysis of variance with Fisher's least significant difference test. The data presented are from two independent experiments. MMC, mitomycin C; DOX, doxorubicin; TXL, paclitaxel; TXT, docetaxel.

Treatment of BT-474 cells with DOX, MMC, or TXT alone, inhibited tumor growth, whereas treatment with TXL caused less inhibition of tumor growth compared with control tumor volumes. However, combined treatment with AS *Bcl-2 *and various anticancer agents caused marked inhibition of tumor growth (Fig. 6b). Statistical analysis showed significant enhancement of the antitumor effect by combinations of AS *Bcl-2 *and anticancer drugs in the treatment group (*P *< 0.05, ANOVA with Fisher's least significant difference (LSD) test). Similarly, combined treatment of ZR-75-1 cells with anticancer drugs and AS *Bcl-2 *also enhanced the antitumor effects of MMC, DOX, TXT, and TXL (Fig. 7). AS *Bcl-2 *statistically enhanced sensitivities to all of the chemotherapeutic agents (*P *< 0.05, ANOVA with Fisher's LSD test). None of the mice treated with AS *Bcl-2 *and anticancer drugs displayed any signs of toxicity. The toxicity of the combined treatments with AS *Bcl-2 *and various anticancer drugs was assessed by comparing weight loss between treated and untreated mice. The weight loss in treated mice was less than 10% (data not shown).

### Effects of combined treatment with AS *Bcl-xL *and various anticancer drugs *in vivo*

**Figure 8:**
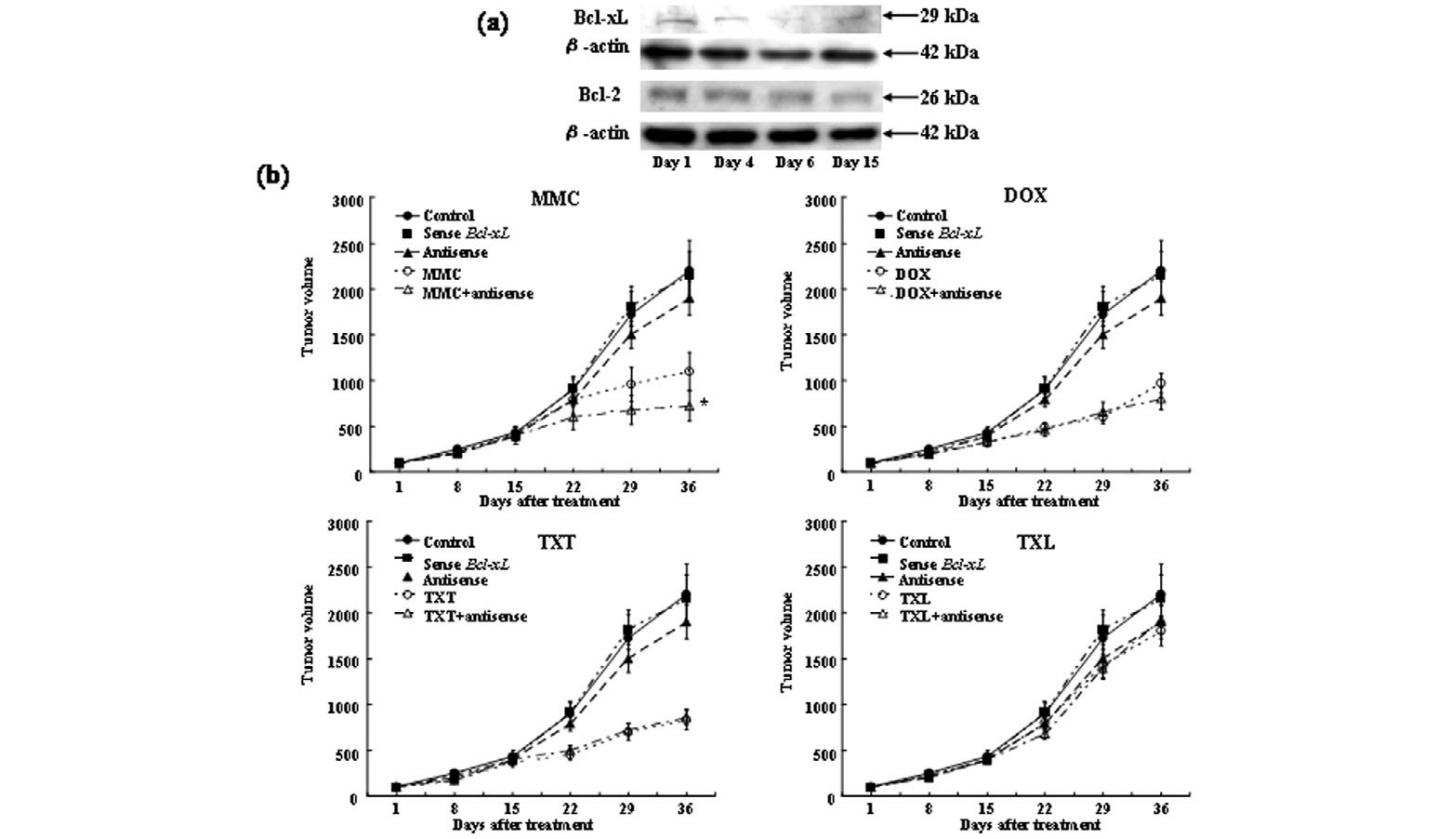
Effects of treatment with antisense *Bcl-xL *and mitomycin C, doxorubicin, paclitaxel, or docetaxel on MDA-MB-231 cells. **(a) **Expression levels of Bcl-xL and Bcl-2 protein in MDA-MB-231 cells transplanted into athymic mice after treatment with antisense (AS) *Bcl-xL *oligodeoxynucleotides (ODNs) were measured by Western blot analysis at the indicated time points. **(b) **Enhancement of the antitumor effects of anticancer drugs by AS *Bcl-xL *ODNs in MDA-MB-231 tumor xenografts. Each point represents the mean tumor volume of the four mice in each group. Error bars indicate SD. *, *P *< 0.05, analysis of variance with Fisher's least significant difference test. The data presented are from two independent experiments. MMC, mitomycin C; DOX, doxorubicin; TXL, paclitaxel; TXT, docetaxel.

The effects of combined treatment with AS *Bcl-xL *and various anticancer drugs were investigated with MDA-MB-231 cells transplanted into athymic mice. As shown in Fig. 8a, Bcl-xL expression was decreased on day 4, further decreased on day 6, and remained low 15 days after treatment with AS *Bcl-xL*. In contrast, Bcl-2 did not change after AS *Bcl-xL *treatment. Treatment with AS *Bcl-xL *alone inhibited tumor growth; however, AS *Bcl-xL *did not enhance the antitumor effects of anticancer agents in MDA-MB-231 cells, apart from MMC (Fig. 8b). Statistical differences in tumor growth were not observed in the combined and regular treatment groups, except for MMC. Combined treatment with AS *Bcl-xL *and MMC significantly enhanced the antitumor effect of MMC (*P *< 0.05, ANOVA with Fisher's LSD test). None of the mice treated with AS *Bcl-xL *and anticancer drugs displayed any signs of toxicity. The weight loss in treated mice was less than 10% (data not shown).

### Immunostimulatory and antitumor effects of AS *Bcl-2 *compared with AS *Bcl-2 *with methylated CpG motifs

**Figure 9:**
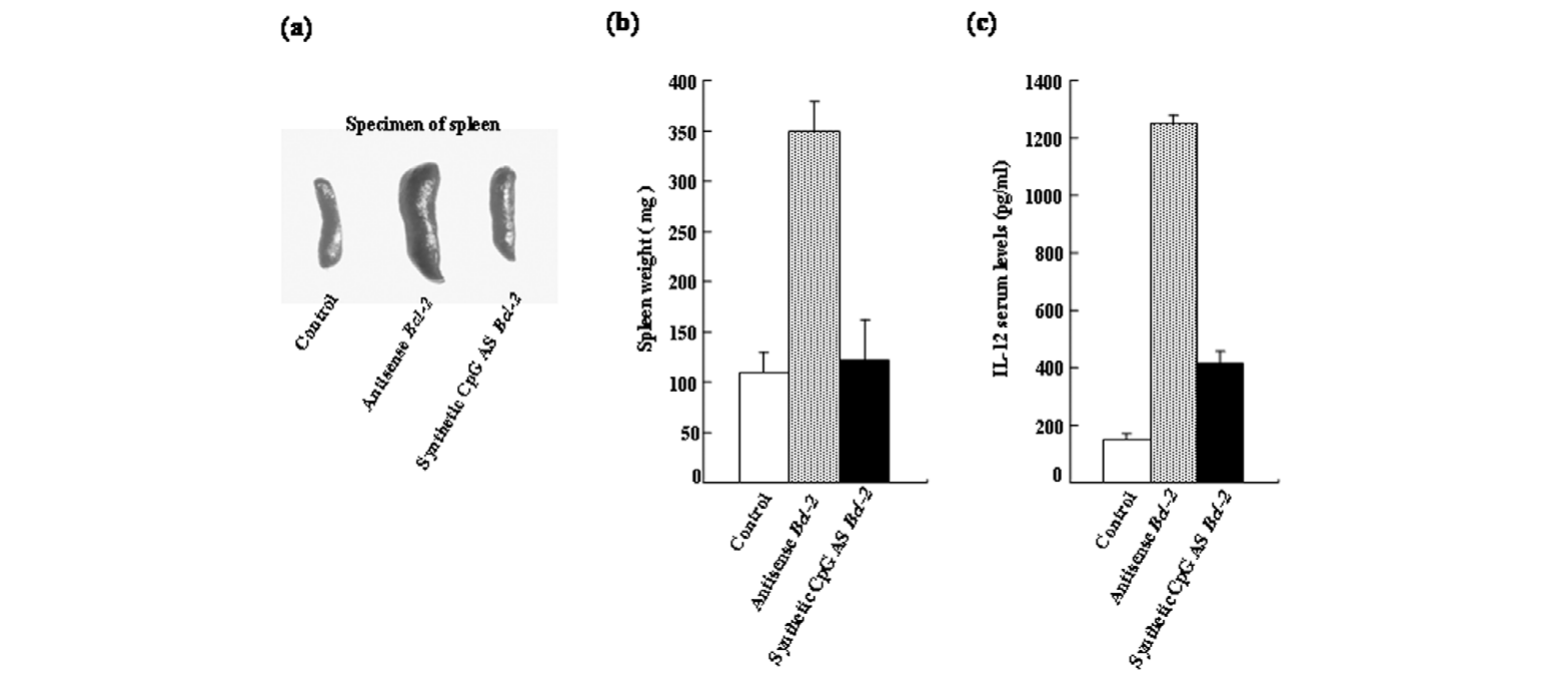
Effect of antisense *Bcl-2 *oligodeoxynucleotides on splenomegaly development and serum IL-12 levels in athymic mice. **(a) **Segment of spleen from a mouse with splenomegaly after treatment with antisense (AS) *Bcl-2 *oligodeoxynucleotides (ODNs), and segments from untreated (control) and synthetic CpG AS *Bcl-2 *ODN-treated mice. **(b) **Comparison of splenic weight among mice treated with AS *Bcl-2 *ODNs, synthetic CpG AS *Bcl-2 *ODNs, and control mice. **(c) **Increased serum levels of IL-12 were observed in mice treated with AS *Bcl-2 *ODNs, compared with those treated with synthetic CpG AS *Bcl-2 *ODNs, and with control mice. Each point represents the mean of the three mice in each group. Error bars indicate SD. The data presented are from three independent experiments.

**Figure 10:**
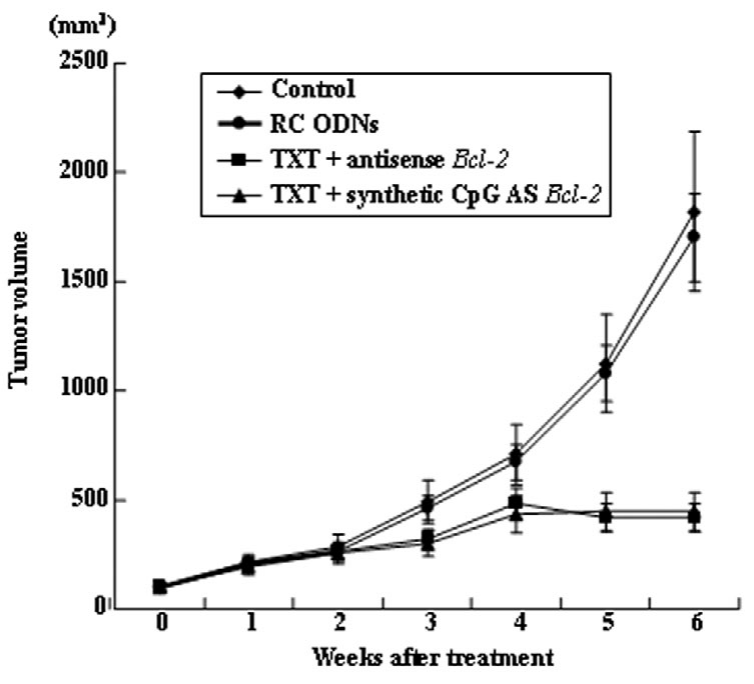
Effect of synthetic CpG antisense *Bcl-2 *on BT-474 cells in comparison with antisense *Bcl-2*. Each point represents the mean tumor volume of the four mice in each group. Error bars indicate SD. The data presented are from two independent experiments. RC, random control; TXT, docetaxel.

To evaluate the possible immunostimulatory role of the CpG motifs of AS *Bcl-2*, we compared the effects of treatment with AS *Bcl-2 *with treatment with synthetic CpG AS *Bcl-2*, in which there is 5'-methylation of the CpG-motif cytosine residues. Mice were treated for 14 days by repeated daily bolus injections of 5 mg/kg AS *Bcl-2*, synthetic CpG AS *Bcl-2*, or saline control. Mice treated with AS *Bcl-2 *demonstrated greater IL-12 levels and splenomegaly, compared with the mice treated with saline and with synthetic CpG AS *Bcl-2 *(Fig. 9). Differences in IL-6 and IFN-γ levels were not observed in mice treated with AS *Bcl-2*-treated and with synthetic CpG AS *Bcl-2 *(data not shown). The antitumor effect of methylated AS *Bcl-2 *was evaluated in BT-474 cells, and the results of treatment with TXT and AS *Bcl-2 *were compared with those of treatment with TXT and synthetic CpG AS *Bcl-2*. TXT was given intraperitoneally once a week for 4 weeks, and AS *Bcl-2 *was given intraperitoneally 6 days a week, every other week for 4 weeks. As shown in Fig. 10, treatment with TXT and AS *Bcl-2*, as well as that with TXT and synthetic CpG AS *Bcl-2*, resulted in enhanced sensitivity to TXT. In addition, no differences in the antitumor effects of AS *Bcl-2 *and synthetic CpG AS Bcl-2 were observed.

## Discussion

In the present study we showed that treatment with AS *Bcl-2 *and AS *Bcl-xL *ODNs produced sequence-specific decreases in protein levels, thereby enhancing the chemosensitivity of BT-474, ZR-75-1, and MDA-MB-231 breast cancer cells to various anticancer drugs both *in vitro *and *in vivo*. Treatment with AS *Bcl-2 *caused greater enhancement of chemosensitivity than treatment with AS *Bcl-xL*. A number of factors might explain the different effects of AS *Bcl-2 *and AS *Bcl-xL *on chemosensitivity, despite the fact that both Bcl-2 and Bcl-xL inhibit apoptotic cell death through the mitochondrial pathway. One determining factor might be the differential expression of Bcl-2 and Bcl-xL in breast cancer cells. Overexpression of Bcl-2 is observed more frequently than overexpression of Bcl-xL (70% versus 40%) in breast cancer tissue, which suggests a more important role for Bcl-2 in conferring drug resistance.

Another influencing factor might be a difference in the ability of sequence-specific AS ODNs to suppress Bcl-2 and Bcl-xL expression. Although our results indicate that greater suppression of Bcl-2 than Bcl-xL was achieved, the *in vitro *and *in vivo *data obtained in this study suggest that differences in Bcl-2 and Bcl-xL suppression do not fully explain their differing effects on chemosensitivity. The differential effects of Bcl-2 and Bcl-xL on drug sensitivity might be unique to breast cancer. In addition, despite similarities in function in the Bcl-2 family proteins, there is evidence to suggest that Bcl-2 and Bcl-xL are subject to different regulatory mechanisms. Bcl-2 inhibits Bid-induced apoptosis at the mitochondrial level by blocking cytochrome *c *release, whereas Bcl-xL does not affect the insertion of tBid into mitochondrial membranes [23,24]. Some reports suggest that Bcl-xL, but not Bcl-2, is capable of modulating apoptosis induced by tumor necrosis factor-related apoptosis ligand (TRAIL) [25].

With regard to the effects of AS *Bcl-2 *on chemosensitivity, sensitivity to DOX and taxanes *in vitro *was increased to a greater extent than sensitivity to MMC in BT-474, ZR-75-1, and MDA-MB-231 cells. Moreover, enhanced sensitivity to DOX and taxanes was more pronounced in BT-474 cells than in ZR-75-1 cells. These findings suggest that the downregulation of Bcl-2 expression by AS *Bcl-2 *enhances drug sensitivity by modulating the apoptotic signal transduction pathway of Bcl-2. The apoptotic signal transduction pathway commonly induced by anticancer agents is associated with the induction of Bax and cleaved PARP, and the downregulation of Bcl-2 and pAkt. Bcl-2 expression is regulated by the ER-responsive element of the promoter region of the *bcl-2 *gene [26], such that overexpression of Bcl-2 might be expected to confer greater drug resistance on ER-positive breast cancer cells. Increases in chemosensitivity to DOX and taxanes *in vitro *did not correlate well with antitumor activity *in vivo*, suggesting that other factors might influence the response of athymic mice to chemotherapeutic agents. The converse was observed for combined treatment of MDA-MB-231 cells with AS *Bcl-xL *and MMC, for which far greater efficacy was observed *in vivo *than *in vitro*.

The role of Bcl-2 in determining the chemosensitivity of breast cancer cells was tested in MDA-MB-453 cells expressing low levels of Bcl-2. Transfection of the *bcl-2 *gene into MDA-MB-453 cells decreased their sensitivity to DOX and MMC but not to taxanes such as TXL and TXT. Several studies indicate that *in vitro *treatment with taxanes induces phosphorylation and inactivation of the Bcl-2 protein as well as apoptosis [27], which might explain why Bcl-2-transfected cells retained their sensitivity to taxanes in the present experiment. Because the enforced overexpression of Bcl-2 can act as an antioxidant in response to DNA damage, the decreased chemosensitivity of the Bcl-2-transfected breast cancer cells to DNA-damaging agents, including DOX and MMC, may be explained by this effect.

Akt is another antiapoptotic protein that belongs to the serine/threonine kinase family. Bcl-2 is activated by Akt through a cyclic-AMP-responsive element-binding protein (CREB) [28]. An Akt- and Bcl-2-dependent pathway might mediate the prevention of anticancer drug-induced cell death through CREB and NF-κB. Because downregulation of Bcl-2 by AS *Bcl-2 *might result in downregulation of pAkt, downregulation of pAkt after treatment with DOX might be augmented by concurrent treatment of ZR-75-1 cells with AS *Bcl-2*.

Previous reports have suggested that AS ODNs have immunostimulatory effects due to their CpG motifs, in addition to their AS activity [29]. In the present study we observed immune stimulation by CpG motifs, resulting in significant spleen enlargement and elevated serum IL-12 levels. However, the role of immune stimulation in mediating antitumor activity remains uncertain because AS *Bcl-2 *ODNs with methylated CpG motifs, when transplanted into athymic mice, demonstrated antitumor activities that were similar to those of their nonmethylated counterparts with immunostimulatory activity. We therefore suggest that the therapeutic activity of AS *Bcl-2 *is due to the AS–mRNA interaction and not to immunostimulatory effects in this model. Similarly, methylated AS *Bcl-2*, with 5'-methylation of CpG motif cytosine residues, had the same antitumor effect as unmethylated AS *Bcl-2 *ODNs in human melanoma xenografts transplanted into mice with severe combined immunodeficiency [30]. However, given that AS *Bcl-2 *stimulates IL-12 secretion and results in the development of splenomegaly, effects that are not observed with methylated AS *Bcl-2*, Th1-mediated immunostimulation may have antitumor effects in solid tumors in humans. Further studies are required to determine whether the immunostimulatory effects of the CpG motifs in AS *Bcl-2 *have antitumor effects in the clinical setting.

Phase III clinical trials using Genasense (known as G3139) in the treatment of patients with chronic lymphocyctic leukemia, malignant melanoma, and multiple myeloma are complete, and are nearly complete for non-small cell lung cancer [31]. Although a phase III trial of G3139/dacarbazine versus dacarbazine alone in advanced malignant melanoma does not show a significant increase in overall survival by the addition of G3139, combination treatment with G3139 and dacarbazine shows a significant increase in progression-free survival and response rate, compared with dacarbazine alone [32]. The results of other phase III trials are eagerly anticipated. In addition, phase I and II trials of G3139 in advanced esophageal, gastric, and colon cancer are ongoing, as are trials in hepatocellular carcinoma, metastatic breast cancer, and hormone refractory prostate cancer.

## Conclusion

Although AS therapy targeting Bcl-2 and Bcl-xL enhances chemosensitivity in breast cancer cells, the effect of blocking Bcl-2 seems superior to that of Bcl-xL. Downregulation of Bcl-2 is associated with enhancement of chemosensitivity to agents such as DOX and taxanes. AS *Bcl-2*-mediated downregulation of Bcl-2 augments anticancer drug-induced signal transduction pathways leading to apoptosis, which is associated with the activation of proapoptotic proteins such as Bax and the suppression of antiapoptotic proteins such as Bcl-2 and pAkt. Although AS *Bcl-2 *ODNs induced splenomegaly and increased serum IL-12 levels in the present experiment, suggesting an immunostimulatory effect of AS *Bcl-2*, the antitumor effect of AS *Bcl-2 *ODNs seemed to result from downregulation of Bcl-2, independently of CpG-mediated immune stimulation.

## Abbreviations

ANOVA = analysis of variance; AS = antisense; CREB = cyclic-AMP-responsive element-binding protein; DMSO = dimethyl sulfoxide; DOX = doxorubicin; ER = estrogen receptor; IL = interleukin; LSD = least significant difference; MM = mismatch control; MTT = 3-(4,5-dimethylthiazol-2-yl)-2,5-diphenyl-2*H*-tetrazolium bromide; ODN = oligodeoxynucleotide; PARP = poly(ADP-ribose) polymerase; RC = random control; TXL = paclitaxel; TXT = docetaxel; VDAC = voltage-dependent anion channel.

## Competing interests

The authors declare that they have no competing interests.

## Authors' contributions

ME carried out the study design, animal feeding, data collection (Western blotting, drug sensitivity assay *in vitro* and *in vivo*, transfection, ELISA), statistical analysis, data interpretation, manuscript preparation, and literature search. RK participated in the design and coordination of the study, in the data interpretation, and manuscript preparation. KT and YU participated in the data collection (Western blotting, transfection). TT organized the study as the director, manuscript preparation, and funding the collection. All authors read and approved the final manuscript.

## Figures and Tables

**Table 1:** Effect of antisense *Bcl-2 *oligodeoxynucleotides on chemosensitivity in BT-474, ZR-75-1, and MDA-MB-231 breast cancer cells

Cell line	Drug	IC_50 _(μM)^a^			Fold increase in AS *Bcl-2*
			
		Control	Sense *Bcl-2*^b^	AS *Bcl-2*^b^	
BT-474	AS *Bcl-2*	3.6	--	--	--
	DOX	1.0	0.97	0.12	8.3
	MMC	1.4	1.3	0.81	1.7
	TXL	2.4	2.3	0.29	8.3
	TXT	0.54	0.50	0.054	10.0
ZR-75-1	AS *Bcl-2*	1.8	--	--	--
	DOX	0.54	0.48	0.075	7.2
	MMC	0.84	0.77	0.33	2.5
	TXL	1.9	1.7	0.62	3.1
	TXT	3.1	2.8	0.80	3.9
MDA-MB-231	AS *Bcl-2*	5.2	--	--	--
	DOX	0.19	0.17	0.032	5.9
	MMC	0.68	0.62	0.22	3.1
	TXL	2.8	2.6	0.43	6.5
	TXT	1.2	1.1	0.23	5.2

AS, antisense; DOX, doxorubicin; IC_50_, concentration for 50% inhibition; MMC, mitomycin C; TXL, paclitaxel; TXT, docetaxel.

^a^The data are the means of three separate experiments (SD < 5%); ^b^cells were treated with 1 μM sense *Bcl-2 *or antisense *Bcl-2*.

**Table 2:** Effect of antisense *Bcl-xL *oligodeoxynucleotides on chemosensitivity in BT-474, ZR-75-1, and MDA-MB-231 breast cancer cells

Cell line	Drug	IC_50 _(μM)^a^			Fold increase in AS *Bcl-xL*
			
		Control	Sense *Bcl-xL*^b^	AS *Bcl-xL*^b^	
BT-474	AS *Bcl-xL*	4.0	--	--	--
	DOX	1.0	0.92	0.68	1.5
	MMC	1.4	1.32	0.88	1.6
	TXL	2.4	2.2	1.34	1.8
	TXT	0.54	0.51	0.26	2.1
ZR-75-1	AS *Bcl-xL*	1.8	--	--	--
	DOX	0.54	0.51	0.71	1.3
	MMC	0.84	0.79	0.39	2.2
	TXL	1.9	1.8	1.2	1.6
	TXT	3.1	3.0	1.7	1.8
MDA-MB-231	AS *Bcl-xL*	2.2	--	--	--
	DOX	0.19	0.18	0.076	2.5
	MMC	0.68	0.65	0.19	3.6
	TXL	2.8	2.6	0.96	2.9
	TXT	1.2	1.1	0.62	1.9

AS, antisense; DOX, doxorubicin; IC_50_, concentration for 50% inhibition; MMC, mitomycin C; TXL, paclitaxel; TXT, docetaxel.

^a^The data are the means of three separate experiments (SD < 5%); ^b^cells were treated with 1 μM sense *Bcl-xL *or antisense *Bcl-xL*.
