# Correction to: Targeted therapy against Bcl-2-related proteins in breast cancer cells

**DOI:** 10.1186/s13058-019-1105-4

**Published:** 2019-02-17

**Authors:** Manabu Emi, Ryungsa Kim, Kazuaki Tanabe, Yoko Uchida, Tetsuya Toge

**Affiliations:** 1 Department of Surgical Oncology, Research Institute for Radiation Biology and Medicine, Hiroshima University, Hiroshima, Japan; 2 International Radiation Information Center, Research Institute for Radiation Biology and Medicine, Hiroshima University, Hiroshima, Japan

**Correction to: Breast Cancer Res**

**https://doi.org/10.1186/bcr1323**

After the publication of this work [1] errors were noticed in Figs. 1a, 6a, and 8a—in which the β-actin bands were mistakenly presented. The corrected Figs. 1, 6, and 8 are presented below. The correction does not affect our conclusions. Nevertheless, we apologize for this error.

**Fig. 1:**
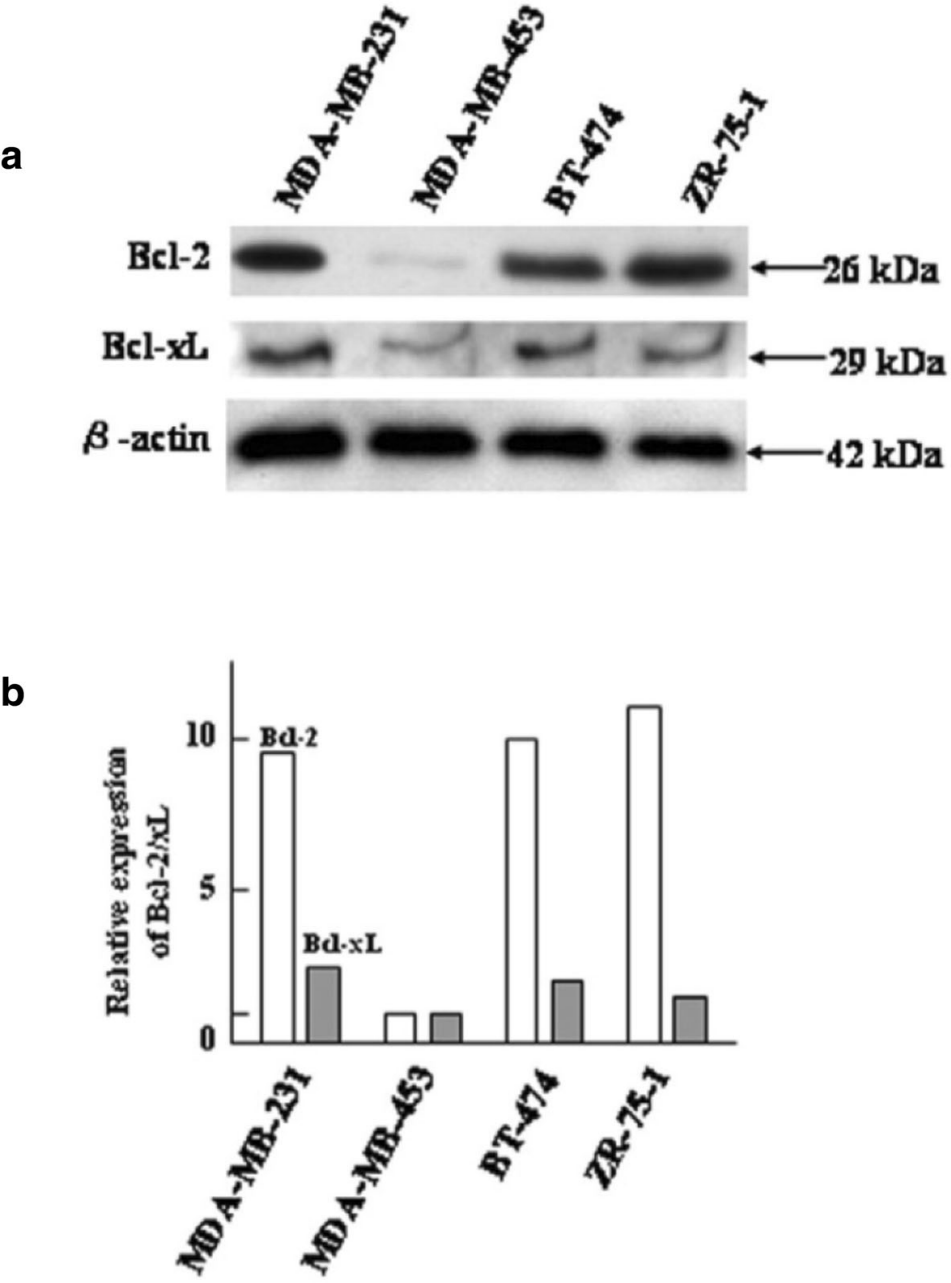
Expression levels of Bcl-2 and Bcl-xL proteins in MDA-MB-231, MDA MD-453, BT-474, and ZR-75-1 cells. **a** Western blot analysis of Bcl-2 and Bcl-xL expression. **b** Quantification of Bcl-2 and Bcl-xL expression by densitometric analysis. The relative expression of Bcl-2 and BclxL in MDA-MB-453 cells was compared with the expression in MDAMB-231, BT-474, and ZR-75-1 cells. Results are from two representative, independent experiments

**Fig. 6:**
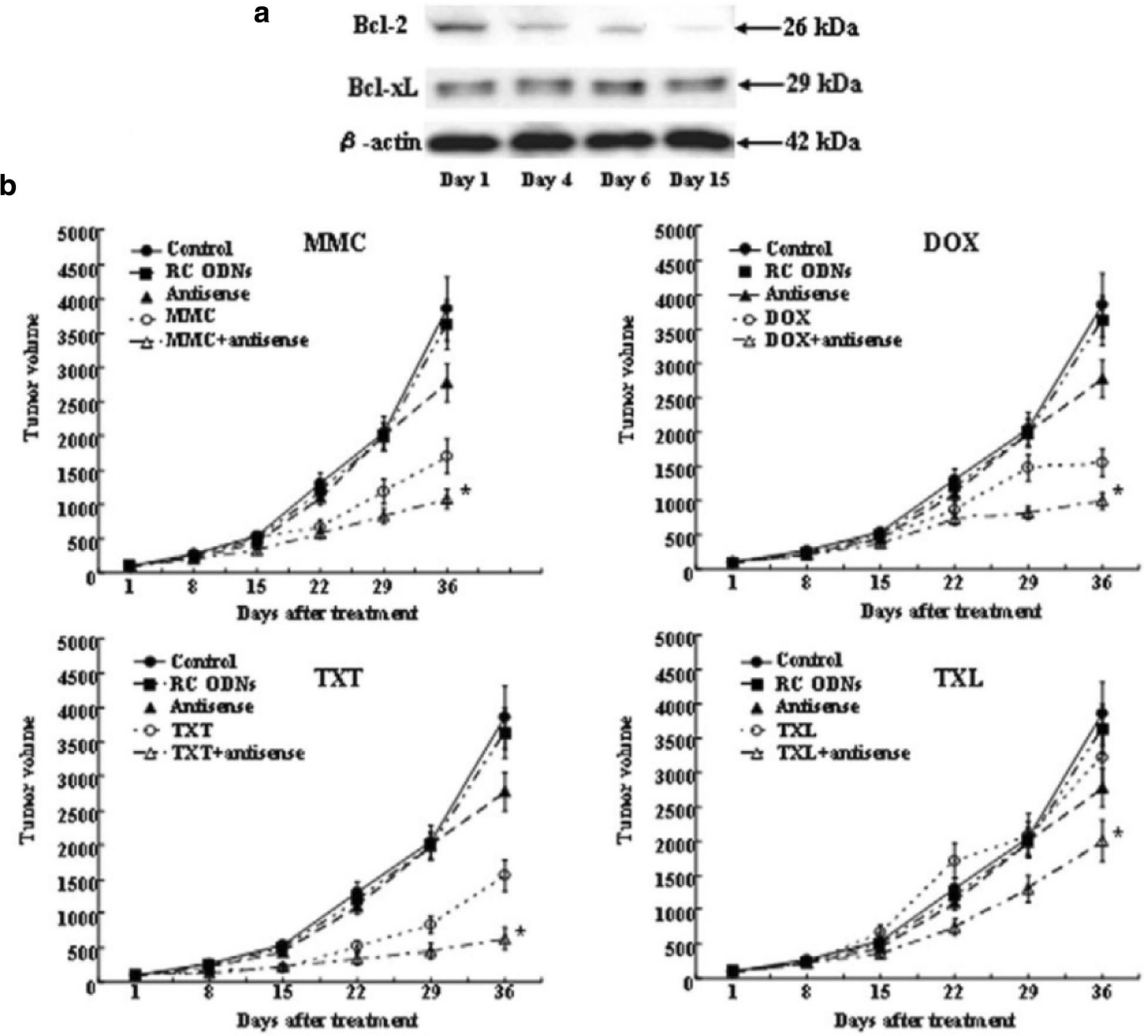
Effects of treatment with antisense *Bcl-2* and mitomycin C, doxorubicin, paclitaxel, or docetaxel on BT-474 cells. **a** Expression levels of Bcl-2 and Bcl-xL protein in BT-474 cells transplanted into athymic mice after treatment with antisense (AS) *Bcl-2* oligodeoxynucleotides (ODNs) were measured by Western blot analysis at the indicated time points. **b** Enhancement of the antitumor effects of anticancer drugs by AS *Bcl-2* ODNs in BT-474 tumor xenografts. Each point represents the mean tumor volume of the eight mice in each group. Error bars indicate SD. *, *P* < 0.05, analysis of variance with Fisher's least significant difference test. The data presented are from two independent experiments. MMC, mitomycin C; DOX, doxoru- bicin;TXL, paclitaxel; TXT, docetaxel

**Fig. 8:**
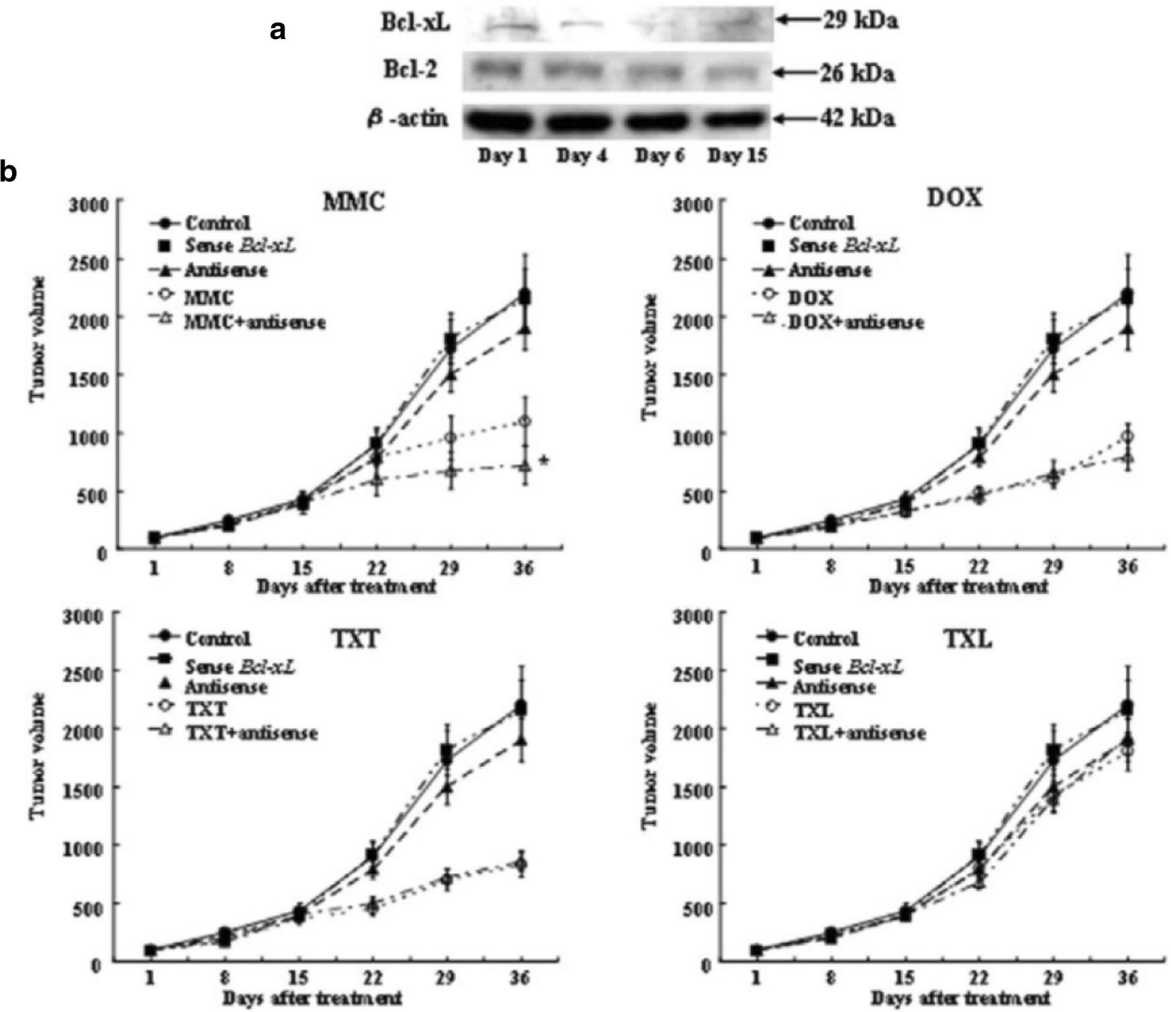
Effects of treatment with antisense *Bcl-xL* and mitomycin C, doxorubicin, paclitaxel, or docetaxel on MDA-MB-231 cells. **a** Expression levels of Bcl-xL and Bcl-2 protein in MDA-MB-231 cells transplanted into athymic mice after treatment with antisense (AS) *Bcl-xL* oligodeoxynucleotides (ODNs) were measured by Western blot analysis at the indicated time points. **b** Enhancement of the antitumor effects of anticancer drugs by AS *Bcl-xL* ODNs in MDA-MB-231 tumor xenografts. Each point represents the mean tumor volume of the four mice in each group. Error bars indicate SD. *, *P*< 0.05, analysis of variance with Fisher's least significant difference test. The data presented are from two independent experiments. MMC, mitomycin C; DOX, doxorubicin; TXL, paclitaxel; TXT, docetaxel
